# Liquid platelet-rich fibrin produced via horizontal centrifugation decreases the inflammatory response and promotes chondrocyte regeneration *in vitro*

**DOI:** 10.3389/fbioe.2023.1301430

**Published:** 2023-12-07

**Authors:** Huimin Li, Ting Xia, Hao Zeng, Yun Qiu, Yan Wei, Yihong Cheng, Yulan Wang, Xiaoxin Zhang, Jin Ke, Richard Miron, Qing He

**Affiliations:** ^1^ State Key Laboratory of Oral and Maxillofacial Reconstruction and Regeneration, Key Laboratory of Oral Biomedicine Ministry of Education, Hubei Key Laboratory of Stomatology, School and Hospital of Stomatology, Wuhan University, Wuhan, China; ^2^ Department of Oral and Maxillofacial Surgery, School and Hospital of Stomatology, Wuhan University, Wuhan, China; ^3^ Department of Periodontology, School of Dental Medicine, University of Bern, Bern, Switzerland

**Keywords:** liquid platelet-rich fibrin, horizontal centrifugation, proliferation, regeneration, chondrocytes

## Abstract

**Objective:** Recently, liquid platelet-rich fibrin (PRF), a rich source of concentrated platelets and growth factors, has emerged as a promising agent for stimulating tissue regeneration. However, its specific efficacy in chondrocyte proliferation and cartilage regeneration remains underexplored. To address this question, we investigated liquid PRF obtained through horizontal centrifugation and compared its effects with hyaluronic acid (HA), a high molecular weight glucosamine supplement widely used in clinical practice to safeguard against chondral damage.

**Materials and Methods:** Liquid PRF, produced using horizontal centrifugation (liquid H-PRF) at 500 g for 8 min, served as our experimental agent. We conducted cell viability and proliferation assays using PRF-conditioned medium. We assessed the chondrocyte phenotype of ATDC5 cells through toluidine blue and alcian blue staining, real-time polymerase chain reaction (RT-PCR), Western blotting, and immunofluorescence staining. Furthermore, we examined the expression of genes involved in inflammation through RT-PCR and Western blot analysis.

**Results:** Liquid H-PRF exerted notable effects on chondrocytes, influencing proliferation, inflammatory responses, and chondrogenic differentiation. The H-PRF group displayed significantly higher expression of chondrogenic markers, including Col2a1, compared to HA-treated cells, whereas aggrecan expression was significantly higher in the HA group. PRF also demonstrated the ability to reduce inflammatory levels in chondrogenic ATDC5 cells, and this effect was further enhanced when PRF from the buffy coat zone was added. In comparison, chondrocytes cultured in the HA group produced significantly fewer inflammatory factors than those in the PRF group, as confirmed qualitatively by Western blot analysis.

**Conclusion:** Liquid H-PRF emerged as a potent stimulator for chondrogenesis and a regulator of the inflammatory response, achieving levels similar to HA. Moreover, liquid H-PRF exhibited strong potential for enhancing the production of cartilage extracellular matrix and promoting chondrogenic regeneration with notably increased Col2a1 levels. Future research should encompass animal studies and human trials to further evaluate the comparative effectiveness of liquid PRF *versus* HA, potentially as an alternative or complementary strategy for future clinical applications.

## Introduction

Osteoarthritis (OA) is a complex and debilitating chronic disease affecting the bones and joints, characterized by the relentless degradation of articular cartilage. The consequences of OA are profound, profoundly affecting the mobility and overall quality of life of afflicted individuals (Hunter and Bierma-Zeinstra, 2019). One of the inherent challenges in OA is the unique nature of cartilage, a specialized connective tissue that lacks intrinsic self-repair capabilities, thereby rendering it susceptible to limited healing capacity after injuries, and eventually culminate in irreversible damage to the affected joints (Poole et al., 2001).

In the quest for effective non-surgical treatments for OA, the more well-known and commonest intra-articular application of hyaluronic acid (HA) has emerged as a promising intervention (Lo et al., 2003). Clinical evidence has demonstrated its capacity to provide relief. However, HA’s inherent limitations, such as its limited chondrogenic regenerative potential and its inability to effectively combat cartilage degeneration, necessitate a pursuit of more advanced alternatives (Spaková et al., 2012).

To date, platelet-rich plasma (PRP) as a first-generation platelet concentrate, has been shown to improve the biomechanical behavior of cartilage, induce chondrocyte proliferation and promote cartilage repair (Kwon et al., 2012; Mifune et al., 2013; Perdisa et al., 2014). Based on the previous literature, apart from the intra-articular injection of HA, PRP has emerged as a promising therapeutic approach for OA, gaining considerable attention. PRP has demonstrated its efficacy by establishing a three-dimensional network within the joint, comprising fibrin and proteins. This network creates an adhesion microenvironment that proves beneficial for various molecules and cells, as highlighted in previous studies (Anitua et al., 2014). Notably, researchers such as Chuan Ye et al. and Samieirad S et al. have shown that the combination of PRP with HA exhibits superior effectiveness in inhibiting OA inflammation compared to PRP or HA administered individually. Furthermore, this combined approach has been proven to enhance joint function while minimizing adverse reactions (Xu et al., 2021; Asadpour et al., 2022).

Nevertheless, the PRP’s widespread use has been hampered by concerns related to suboptimal optimization and the requirement for anticoagulants (Kobayashi et al., 2016). Consequently, platelet-rich fibrin (PRF), another platelet concentrate, has garnered increased attention due to its documented efficacy across various medical domains. Injectable PRF (i-PRF) notably revolutionized platelet concentrate collection by enabling it to be obtained in liquid form without anticoagulants by using more hydrophobic plastic blood collection tubes, thus opening up numerous opportunities in regenerative medicine (Sordi et al., 2022). Recent prospective studies have highlighted the superior long-term efficacy of PRF compared to HA and PRP. Additionally, PRF has been shown to reduce the risk of potential adverse effects. Nevertheless, further research is needed to comprehensively understand the underlying mechanism (Vingender et al., 2023; Xu et al., 2023).

In 2019, a groundbreaking method emerged, introducing horizontal centrifugation as a novel approach to produce PRF (H-PRF). This technique yielded remarkable results by alleviating platelets loss in the final PRF concentrate, demonstrating an up to fourfold increase in cell concentrations, including platelets and leukocytes, compared to PRF prepared by conventional fixed-angle devices (Feng et al., 2023). Intriguingly, despite these promising advances, no published data exists on the efficiency of liquid H-PRF on cartilage regeneration or chondrocyte regeneration to date. Consequently, this study’s primary objective was to investigate the impact of liquid H-PRF on chondrocyte proliferation and cartilage regeneration. Additionally, we sought to explore its potential role in managing inflammation, comparing its performance to the widely used hyaluronic acid (HA) treatments. This research endeavors to shed light on a potentially therapeutic strategy in the management of OA.

## Materials and methods

### Preparation and isolation of liquid horizontal PRF (H-PRF)

All protocols used in this study were approved by the Ethics Committee of the School and Hospital of Stomatology, Wuhan University (B52/2020). Informed written consents were obtained from 10 healthy volunteers (average age 30 years; age narrow from 22 to 38-years old) with no blood-related disorders who underwent the routine blood draws/collection as previously described (Feng et al., 2022; Feng et al., 2023). The standard or criteria excludes people with systemic rheumatoid disease, cardiovascular disease or coagulation disorders, history of neurologic medication intake, concurrent use of anti-inflammatory drugs due to the fact that they neutralize PRF’s effect, according to previous research (Asadpour et al., 2022). The blood was centrifuged immediately after collection. Approximately 10 mL of venous blood was collected from each volunteer using plastic PET tubes to prepare liquid H-PRF. By using more hydrophobic plastic blood collection tubes without need/addition of any anticoagulants, the liquid H-PRF was prepared using a previous reported protocol utilizing horizontal centrifugation at 500 *g* for 8 min at room temperature (Feng et al., 2022).

### Chondrocytes culture and chondrogenic differentiation

The ATDC5 cell line is characterized as a chondrogenic cell line and purchased from ATCC. In this study, ATDC5 chondrocytes were cultured and passaged in Dulbecco’s Modified Eagle’s Medium/F12 (DMEM/F12) supplemented with 10% fetal bovine serum (FBS), in a humidified incubator with 5% CO_2_ at 37°C. To induce chondrogenic differentiation, the ATDC5 cells were treated with culture medium containing 1% insulin-transferrin-selenium (ITS, Cyagen Biosciences, China) liquid media supplement for a period of 10–14 days. The chondrogenic differentiation medium was replaced every 2–3 days during differentiation.

### PRF conditioned medium and exudate incubation

After 10–14 days chondrogenic differentiation, co-culture experiments of chondrocytes with different H-PRF conditioned medium percentages were conducted in a Transwell assay system (Costar, Cambridge, MA, United States of America) for optimal treatment imitating the intra-articular condition. And the treatment concentration was referred to the previous studies with a final concentration of 2.5% and 5% PRF (Kobayashi et al., 2016; Fujioka-Kobayashi et al., 2017; Zhang et al., 2020). These PRFs were used by single patient or subject without mixing. And each PRFused in the experiment by triplicate replicates. ATDC5 cells were suspended in DMEM with 5% FBS, and then cultured in the lower compartment of the Transwell system. Original PRF collected by a small syringe with an 18G hypodermic needle was prepared by adding chondrocyte medium to achieve a final concentration of 2.5% and 5% PRF. with or without buffy coat (Thanasrisuebwong et al., 2020; Zhang et al., 2020). The prepared PRF medium with 5% FBS was loaded in the upper chamber to observe the effects of PRF culture medium with different components on chondrocytes, and the wells loaded with hyaluronic acid (HA, 100 μg/mL) was served as positive group. All the procedures were conducted before the PRF mixture was solidified. The cells were then cultured for 24 h. After that, the chondrocytes in the lower compartment were collected for RNA or protein, or fixed upon pre-seeded coverslips in 4% paraformaldehyde for subsequent EdU and immunofluorescence staining experiments.

### Cell viability and proliferation assay

Cell viability was assessed using the CCK-8 Cell Counting Kit (Vazyme Biotech, China) following the manufacturer’s instructions (Xu et al., 2016). A total of 2×10^3^ ATDC5s were seeded in 96-well plates and treated with varying doses of PRF for 12–96 h. After incubation, 10 μL CCK8 solution was added to each well and incubated at 37°C for 1 h. The absorbance was then measured at 450 nm using a microplate reader (Biotek, Winooski, United States). To measure cell proliferation, Edu assay was performed using Cell-Light™ EdU Apollo^®^488 *In Vitro* Imaging Kit (Ruibo, Guangzhou, China) according to the manufacturer’s instructions. The number and proportion of EdU incorporated cells were examined and the percentage of positively stained cells was quantified using Image-Pro Plus 6.0 (The National Institute of Health, MD, United States).

### Toluidine blue and alcian blue staining

The expression of Glycosaminoglycans expression was analyzed by alcian blue and toluidine blue staining. Cells were seeded at a density of 5 × 10^4^ per well in a 24-well plate and treated with different PRF groups for 24 h following differentiation induction. After treatment, cells were washed twice with phosphate buffered saline (PBS), fixed with 4% paraformaldehyde, and stained with 1% alcian blue or 0.5% toluidine blue dyeing solution for 1 h at room temperature. The percentage of toluidine blue and alcian blue stained area was measured and quantified by ImageJ.

### Quantitative real-time-PCR analysis

Total RNA was isolated using RNAiso Plus reagent (Invitrogen)and quantified using a Nanodrop spectrophotometer. Reverse transcription was performed using a reverse transcription kit (Vazyme Biotech, China) to generate cDNA. Quantitative real‐time PCR analysis was conducted using a real‐time SYBR PCR kit (Vazyme Biotech, China). The expression levels of target genes were calculated using 2^−ΔΔCT^ formula after normalization to the expression of the housekeeping gene, Glyceraldehyde-phosphate dehydrogenase (GAPDH). The sequences of real time PCR primers were listed in Table 1.

**TABLE 1:** Oligonucleotide primer sequences utilized in this article.

Acan-Forward	GACAAAGACAGCAGCCCAGGAG
Acan--Reverse	CGAGGCGTGTGGCGAAGAAC
Col2a1-Forward	GGGAATGTCCTCTGCGATGAC
Col2a1-Reverse	GAAGGGGATCTCGGGGTTG
Adamts5-Forward	GGCAAATGTGTGGACAAAACTA
Adamts5-Reverse	GAGGTGCAGGGTTATTACAATG
Mmp13-Forward	CTTCCTGATGATGACGTTCAAG
Mmp13-Reverse	GTCACACTTCTCTGGTGTTTTG
Il6-Forward	CTCCCAACAGACCTGTCTATAC
Il6-Reverse	CCATTGCACAACTCTTTTCTCA
Il1b-Forward	CACTACAGGCTCCGAGATGAACAAC
Il1b-Reverse	TGTCGTTGCTTGGTTCTCCTTGTAC
Tnf-Forward	ATGTCTCAGCCTCTTCTCATTC
Tnf-Reverse	GCTTGTCACTCGAATTTTGAGA
COX-2-Forward	ATTCCAAACCAGCAGACTCATA
COX-2-Reverse	CTTGAGTTTGAAGTGGTAACCG

### Western blot analysis

Total protein of chondrocytes was extracted with radioimmunoprecipitation (RIPA) lysis buffer, and subsequently separated by electrophoresis on sodium dodecyl sulfate-polyacrylamide gels (SDS-PAGE), then transferred onto PVDF membranes. After antigen blocking for15 min in a blocking solution (Beyotime, China), the membranes were incubated with the following primary antibodies overnight at 4°C: rabbit anti-Aggrecan (1:1000, Proteintech, 13880-1-AP), rabbit anti-Col2a1 (1:1000, Abclonal, A16891), rabbit anti-Adamts5 (1:1000, abcam, ab41037), rabbit anti-Il-6 (1:1000, Proteintech, 21865-1-AP), mouse anti-Il-1β (1:1000, Abclonal, A16288), mouse anti-GAPDH (1:1000, abcam, ab8245). The membranes were then incubated with HRP conjugated secondary antibodies for 1 h. Finally, protein blotting was visualized by Odyssey^®^ Fc Imaging System (LI-COR, United States of America) after ECL reagent reaction and quantified using its statistical module.

### Immunofluorescence staining and analysis

ATDC5 cells were plated on sterile coverslips and differentiated to subconfluence in a 24-well plate, and then treated as indicated previously for 24 h in a transwell system. After paraformaldehyde fixation, the coverslips were collected and processed for immunofluorescence staining. Harvested cells were permeability enriched with Triton X-100 solution (1%), antigen blocked, then incubated with rabbit anti-Aggrecan antibody (1:500, Proteintech, 13880-1-AP) and anti-Col2a1 antibody (1:500, Abclonal, A16891) overnight at 4°C. On the second day, Dylight 594 Goat anti-Rabbit IgG and Dylight 488 Goat anti-Rabbit IgG were introduced separately and allowed to react with the cells. The nuclei were then stained and sealed with mounting medium containing DAPI. Fluorescence intensity was monitored and visualized under a confocal fluorescence microscope (Stedycon, Nikon, Japan) and analyzed using Image-Pro Plus software.

### Statistical analysis

Each test was repeated at least three times independently and all experimental data are represented as the mean ± SD. Statistical analysis was conducted with one-way ANOVA analysis of variance by using GraphPad Prism 8.0 software. Statistical significance was set at ^∗^ *p* < 0.05, ^∗*^ *p* < 0.01, ^∗**^ *p* < 0.001 compared to control group; ^#^ *p* < 0.05 compared to PRF-BC 5% group.

## Results

### Preparation of liquid H-PRF

**FIGURE 1:**
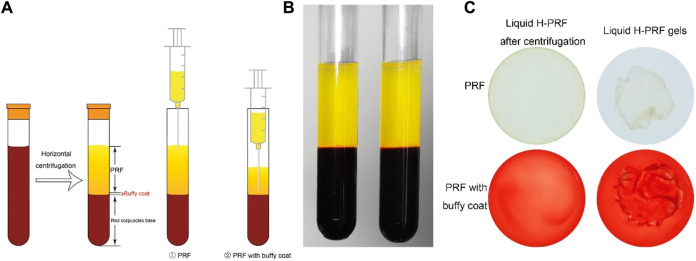
Method to collect and concentrate PRF. **(A)** Schematic illustration of preparation the liquid PRF with or without buffy coat after horizontal centrifugation process. **(B)** The state of liquid PRF after centrifugation. **(C)** Liquid PRF formed into a whole gel in final ready form.

Upon subjecting blood samples to horizontal centrifugation, distinct layers formed, as depicted in Figure 1. A noticeable horizontal separation emerged between the lower layer of red corpuscles and the upper layer containing liquid H-PRF. To collect the liquid H-PRF layer for our PRF group, an 18G hypodermic needle attached to a small syringe was employed, aligning with the methodology established in previous studies (Kargarpour et al., 2020; Kargarpour et al., 2021). Meanwhile, separation liquid H-PRF layer containing the buffy coat zone was specified as the PRF with buffy coat, PRF-BC group for short. The majority of leukocytes and platelets were incorporated within this middle cell-rich layer containing a plasma and RBC component (Fujioka-Kobayashi et al., 2020). When the liquid H-PRF was transferred to 6-well plates with tilting the plates to 45° every 15 s for 20 min, fibrin clot formation was observed. The following experiments with different PRF treatments was prepared before the final solidification time.

### PRF increased ATDC5 proliferation

**FIGURE 2:**
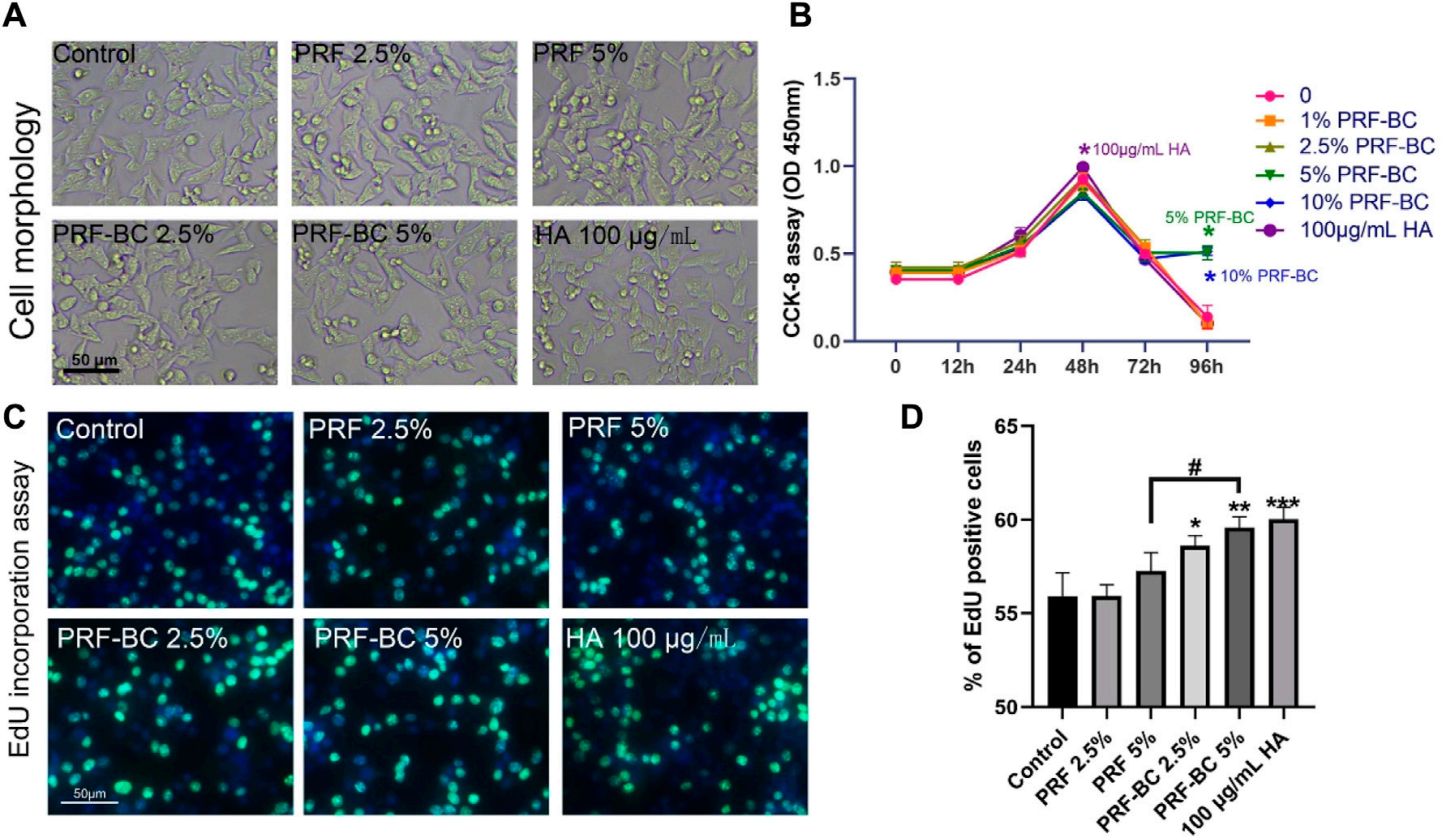
Liquid PRF below the concentration of 5% has no significant suppression on cell morphology, viability, proliferation. Cellular morphology was analyzed and photographed after various treatment under the light microscope with ×10 magnification **(A)**. In contrast, the CCK-8 assay showed PRF and HA did not suppress the cell viability under the working concentration **(B)**. EdU incorporation assays indicated H-PRF with buffy coat promoted the cell proliferation of ATDC5 cells almost as well as HA group **(C,D)**. All data are depicted as mean ± SD (n = 3; ^*^ *p* < 0.05, ^**^ *p* < 0.01, ^***^ *p* < 0.001 compared to control group; ^#^ *p* < 0.05 compared to PRF-BC 5% group).

The ATDC5 cell line exhibits gradual chondrogenic differentiation similar to the development process observed in chondrogenesis. At the concentration less than 5% of PRF, there was no effect on cell viability, number, and morphology (Figure 2A). The viability of ATDC5, as detected by CCK-8 analysis, was not inhibited under the working concentration (Figure 2B). In the 5% and 10% PRF groups, the viability of ATDC5 was slightly elevated compared to the control group only after 96 h of incubation. This could be due to the continued release of greater growth factors in PRF with buffy coat from horizontal centrifugation. In accordance, PRF released the highest total growth factors over time compared to PRP or other low speed horizontal centrifugation exuded PRF (Kobayashi et al., 2016). As growth factors maintained in platelet concentrates have been shown to increase cell viability, proliferation and collagen deposition ability during tissue remodeling and regeneration process. This is why we chose the horizontal centrifugation protocol of 500 g for 8 min to prepare liquid H-PRF. Figures 2C, D showed that PRF increased proliferation, and H-PRF with buffy coat promoted cell proliferation of ATDC5 cells at comparable level to the HA group. In the positive control group of 100 μg/mL HA, the viability and proliferation ability of chondrocytes were increased, and these results are consistent with previous research (Guo et al., 2015; Li et al., 2019).

### Extracellular matrix synthesis level was upregulated with PRF treatment

**FIGURE 3:**
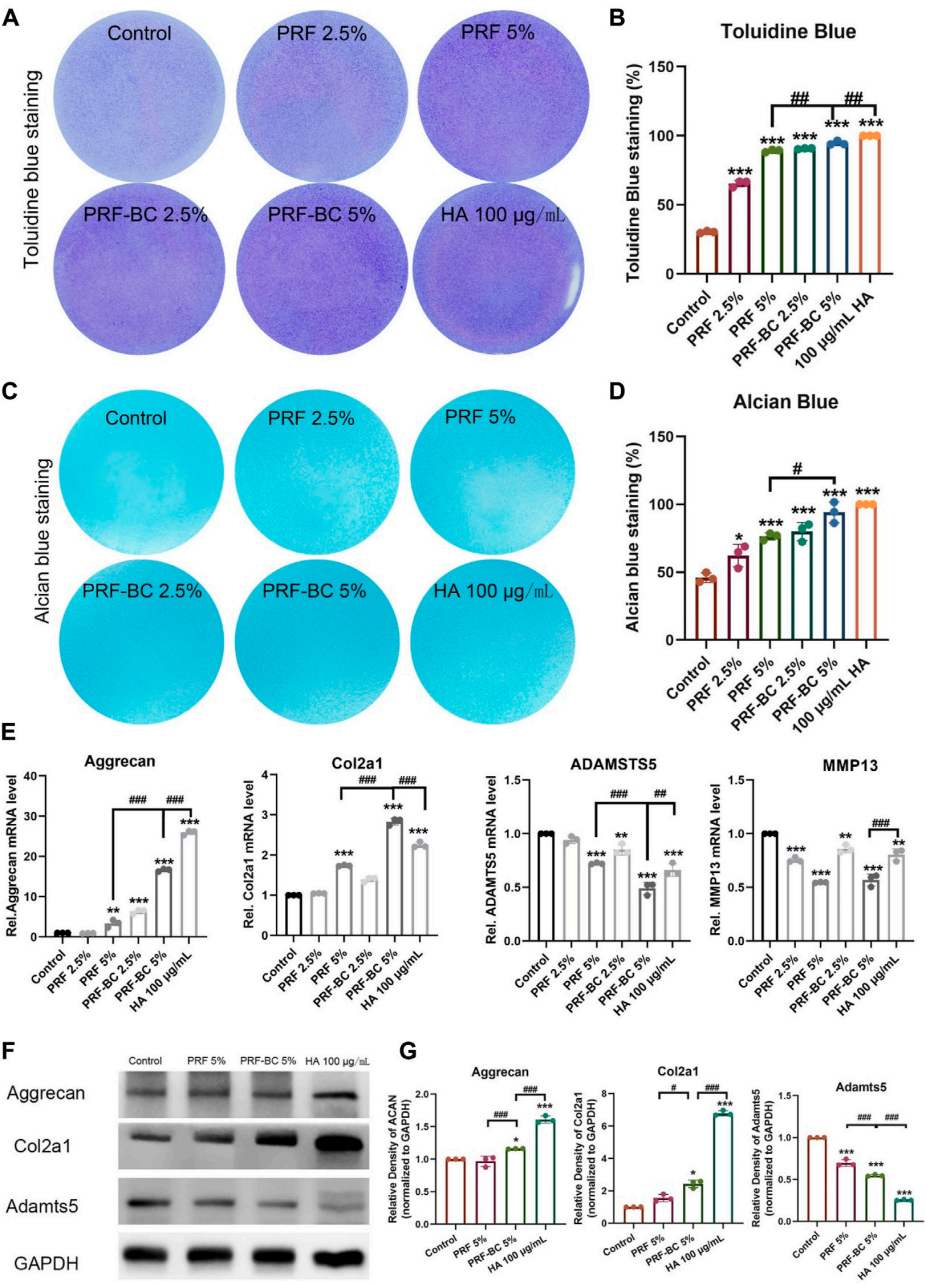
Toluidine blue and alcian blue staining uncovered that PRF treatment amplified extracellular matrix synthesis level of ATDC5 chondrocytes. **(A,B)** Toluidine blue and **(C,D)** alcian blue staining uncovered that PRF treatment induce chondrogenic differentiation. A and C figures display the images of staining, and B, D panels are the quantification of the stainings. **(E)** RT-qPCR analysis revealed PRF promote regeneration in chondrocytes in mRNA level. **(F)** WB analysis demonstrates PRF promoted regeneration in chondrocytes no less than HA group in some indicator. **(G)** Quantification of panel F by ImageJ. All data are depicted as mean ± SD within scatter plots, *n* = 3, ^*^ *p* < 0.05, ^**^ *p* < 0.01, ^***^ *p* < 0.001 compared to control group; ^#^ *p* < 0.05, ^##^ *p* < 0.01, ^###^ *p* < 0.001 compared to PRF-BC 5% group.

Toluidine blue and alcian blue staining demonstrated that PRF, regardless of buffy coat incubation, enhances the extracellular matrix of chondrocytes, which is primarily composed of aggrecan and Col2A1 (Figures 3A–D). These findings suggest that PRF could facilitate anabolic activity in chondrocytes by regulating extracellular matrix homeostasis (Peng et al., 2021). PRF with the combination of buffy coat had greater biological properties when compared to single PRF group. And HA positive group were superior in function of promoting differentiation when compared to all other groups except 5% PRF-BC group both confirmed by toluidine blue and alcian blue staining. Real-time PCR analysis demonstrated that platelet-rich fibrin with buffy coat promotes regeneration in chondrocytes by increasing the RNA levels of anabolic genes, such as Aggrecan, Col2a1 (Figure 3E).

### Anabolic synthesis level was upregulated more by PRF with buffy coat treatment

Based on these results, we selected the groups with the most significant effects for further experimentation. These groups included the PRF 5%, PRF-BC 5%, and 100 μg/mL HA treated groups. Consistent with toluidine blue and alcian blue staining, as well as real-time PCR results, Western blot analysis confirmed that the anabolic mediator aggrecan and Col2a1 was upregulated by PRF or HA treatment. We observed that HA treatment provided a more extended advantage in both aggrecan and Col2a1 accumulation compared to PRF, with or without buffy coat (Figures 3F, G). Moreover, the PRF-BC 5% group also induced the significant Col2a1 expression than PRF without buffy coat group (Figures 3E–G). Same conclusion has been confirmed by cell immunofluorescence analysis, that the anabolic mediator aggrecan and Col2a1 was upregulated by PRF or HA treatment. However, PRF-BC 5% treatment stimulated Col2a1 more significantly (Supplementary Figure S1).

### PRF reduced catabolic level and inflammatory level in ATDC5 cells

**FIGURE 4:**
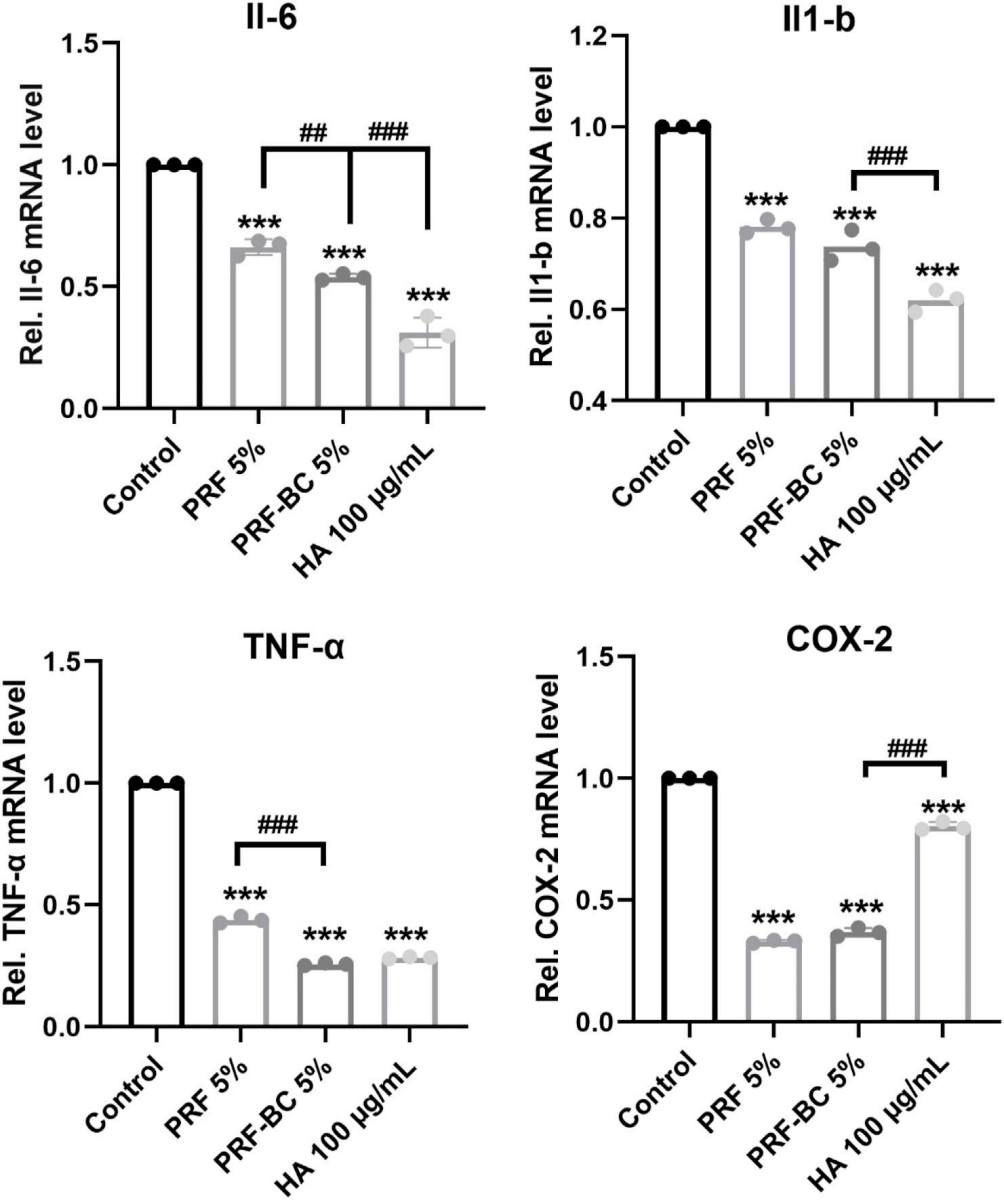
Real-time RT-PCR analysis revealed PRF reduce inflammatory response in chondrocytes, but with less anti-inflammatory capacity than HA group. All data are depicted as mean ± SD (n = 3; ^*^ *p* < 0.05, ^**^ *p* < 0.01, ^***^ *p* < 0.001 compared to control group, and ^#^ *p* < 0.05, ^##^ *p* < 0.01, ^###^ *p* < 0.001 compared to PRF-BC 5% group.).

Real-time PCR analysis revealed PRF or HA treatment also significantly repressed the expression of catabolic mediators including Adamst5 and Mmp13, inhibition of which would protect from osteoarthritis progression (Figure 3E). Moreover, PRF or HA treatment inhibited inflammation. The mRNA expression levels of Il-6, Il-1β, TNF-α, and COX-2 had been significantly repressed by these treatments, and PRF-BC 5% presented similar or milder inhibition effects when compared with HA group (Figure 4).

## Discussion

In our study, liquid H-PRF has been demonstrated as a potent stimulator for chondrogenesis, effectively regulating the inflammatory response and achieving results comparable to HA as an alternative treatment for OA. Particularly, liquid H-PRF has exhibited significant potential in enhancing the production of cartilage ECM and promoting chondrogenic regeneration, leading to notably increased synthetic levels. Moreover, liquid H-PRF exhibits an anabolic effect on chondrocytes by facilitating proliferation, inhibiting degradation, and supporting matrix development, thereby maintaining appropriate chondrocyte growth and development.

Liquid PRF is a completely natural, autologous and unmodified platelet concentrate that does not require any additives or anticoagulants (Miron et al., 2019; Agrawal and Jaiswal, 2020). Its favorable safety profile and minimal manipulation have led to a wide range of studies, including *in vitro*, *in vivo*, and clinical studies, demonstrating its regenerative potential and superior anti-inflammatory ability in various medical and dental applications. Wang Z (Wang et al., 2021) and Lee JS (Lee et al., 2022) et al. have demonstrated that PRP is capable of promoting chondrogenic differentiation of stem cells, which is consistent with our own results. As the final therapeutic conclusion of HA and PRP for the treatment of OA is still undefined throughout so many basic research and preclinical studies (Zhang et al., 2022; Lu et al., 2023), this is a meaningful research direction not only in basic research, but also for future clinical research.

Our study found that PRF treatment upregulated extracellular matrix synthesis levels. However, Lucienne A. Vonk et al. reported that PRP treatment did not induce extracellular matrix synthesis (Rikkers et al., 2021). In their research, they collected PRP but not liquid H-PRF, and their centrifuge protocol was different, with platelets pelleted at 130 × g for 15 min and then further centrifuged at 250 *g* for 15 min, with the pelleted platelets resuspended in one-third of the supernatant platelet-poor plasma (PPP). The release of growth factors and cells such as leukocytes in PPP or PRP was significantly lower than in PRF, and it degenerated faster over time, as previously demonstrated (Kobayashi et al., 2016). PRP formulations contain several variables, with platelet concentration, white blood cell (WBC) concentration, and growth factor amount and quality being the most important categories (Mazzocca et al., 2012). Furthermore, PRF contains more growth factors than PRP, including IGF-I, FGF, EGF, and PDGF, which play important regulatory roles in collagen synthesis and could prevent the change in the ECM molecule caused by osteoarthritis to some extent (Xie et al., 2014; Zhou et al., 2016). This highlights the importance of a modified optimal protocol to preserve the regenerative activity from whole blood, so that biological performance is significantly improved through certification in our experiment.

Compared to the positive clinical use of HA (Gilat et al., 2021), which is a glycosaminoglycan that serves as a critical component of ECM proteoglycans, H-PRF has also been shown to be effective in controlling inflammation. Equivalent to the previous studies results (Marcazzan et al., 2018; Zhao et al., 2021), the H-PRF with buffy coat showed better anti-inflammation efficacy than single PRF group, and furthermore exhibited less relative COX-2 mRNA expression than HA group. Compared to HA group, although it has less influence in reducing inflammation and aggrecan production, it showed relatively more positive effect than single PRF group. The cartilage regenerative ability of 5% PRF with buffy coat is markedly better than PRF without buffy coat group. Our previous research has demonstrated that the red buffy coat layer, which is essential for the adequate concentration of liquid H-PRF, contains the majority of leukocytes (Ghanaati et al., 2014). This is particularly relevant to the better outcome in the PRF-BC group than the PRF without buffy coat group when the liquid H-PRF is used as a bioactive conditioned medium for proliferative and regenerative purposes. Moreover, the PRF-BC group provided long-term improvement in cell viability and proliferation compared to the PRF group. As we just investigated the single effect of PRF or HA, based on Kon et al.‘s study of autologous PRP injection demonstrating longer efficacy than HA injection in relieving symptoms (Chen et al., 2014), it indicates HA-PRF with buffy coat conjugate should be further conducted and may show more promising functionality synergistically for a longer period of time.

Previous studies have demonstrated that high molecular weight HA not only has the ability to suppress inflammation but also inhibits nitric oxide-induced apoptosis and dedifferentiation of articular chondrocytes in osteoarthritis (Peng et al., 2010; Partain et al., 2021). Therefore, HA may play an important role in down-regulating the Il-1β expression and may be more efficient for aggrecan expression than PRF alone. We expect that the efficacy of liquid H-PRF together with HA warrants even better results.

While this study provides valuable insights into the potential benefits of using liquid H-PRF for promoting the regeneration of joint cartilage, it is important to acknowledge some limitations. First, the ATDC5 cell line used in this study is derived from mice and may not be readily available for clinical use in humans. Additionally, ATDC5 cell culture could not mimic the real osteoarthritic cartilage which has very limited regenerative capacity. These could impact the applicability of these findings to human patients. Therefore, further research using human temporomandibular chondrocytes is needed to confirm these results.

Another limitation of this study is the lack of animal studies. Future research should explore the effects of liquid H-PRF on promoting joint cartilage regeneration and anti-inflammatory effect *in vivo*. Although some PRPs as pharmaceutical preparation has conducted in many clinical studies (GöRMELI et al., 2017; Huang et al., 2019), and suggested that multiple PRP injections more than three times are useful in achieving better clinical results and is comparable to HA group in 3 months, the investigation of liquid H-PRF with better performance than traditional PRPs in preclinical and animal studies remains blank. Moreover, the protocol should be standardized to obtain most effectiveness, which could provide a reference for the clinical application of liquid H-PRF in joints. H-PRF combined with HA treatment will be further explored, comparing it with H-PRF or HA administered individually. Until now, there has been limited research directly comparing these treatment groups. Our upcoming investigation aims to delve deeper into the significance of this alternative treatment approach. Overall, while this study is a significant step forward in our understanding of liquid H-PRF’s potential in promoting joint cartilage regeneration, more research is necessary before its clinical efficacy can be fully realized.

## Data Availability

The original contributions presented in the study are included in the article/Supplementary Material, further inquiries can be directed to the corresponding author.
