# Supplementary figures and images for: Liquid platelet-rich fibrin produced via horizontal centrifugation decreases the inflammatory response and promotes chondrocyte regeneration in vitro
Source: Front Bioeng Biotechnol. 2023 Dec 7;11:1301430. doi: 10.3389/fbioe.2023.1301430 (PMC10740190; doi:10.3389/fbioe.2023.1301430)

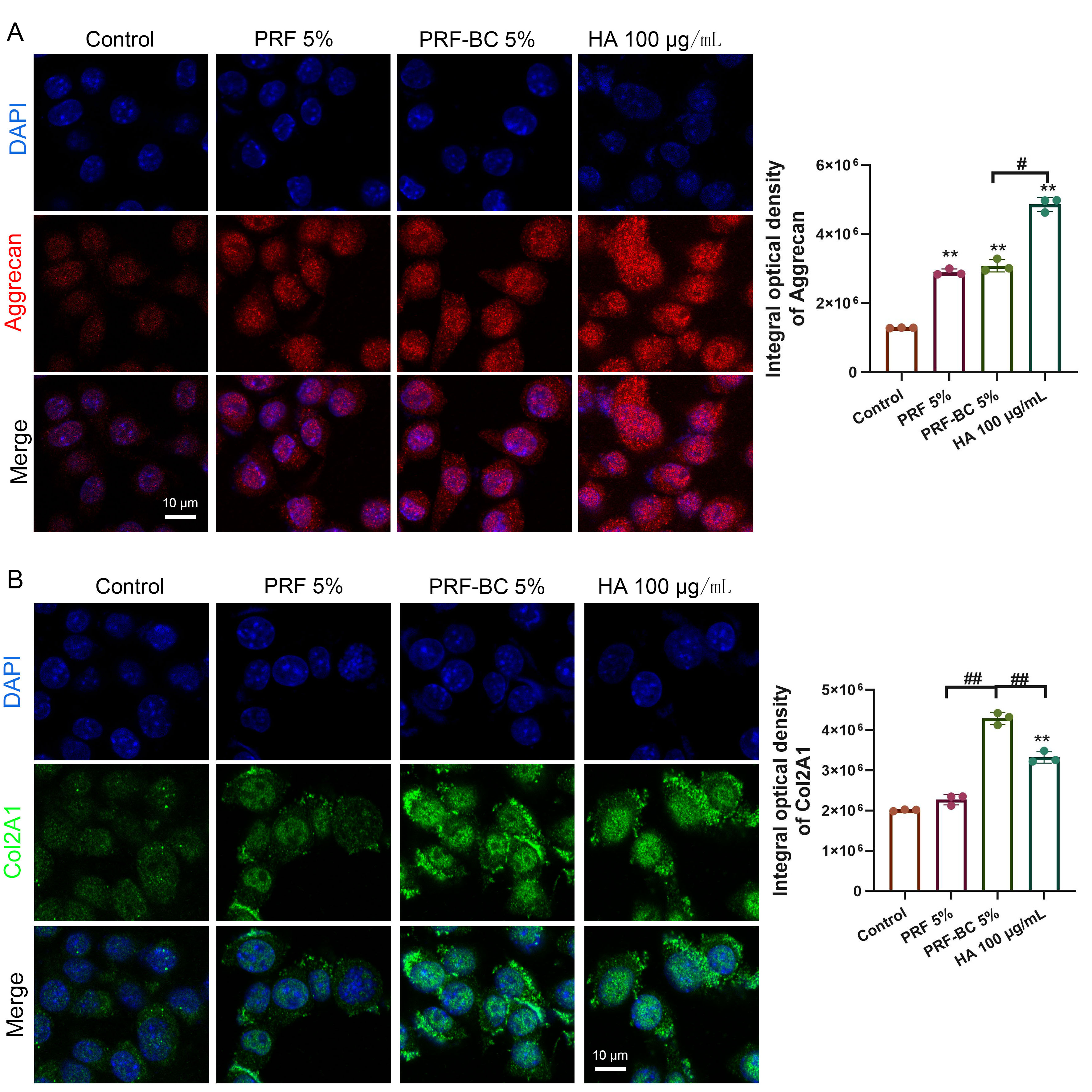

Supplement: Supplementary file 1 [file Image1.JPEG]

Aggrecan

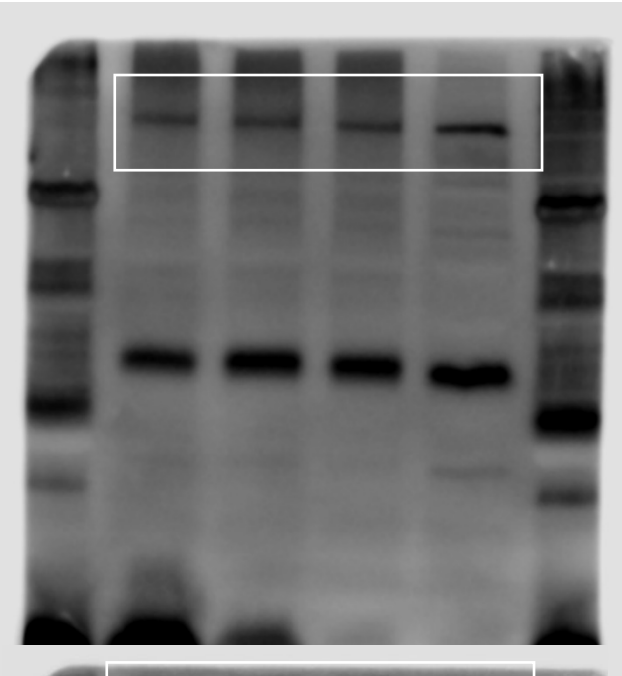

Col2A1

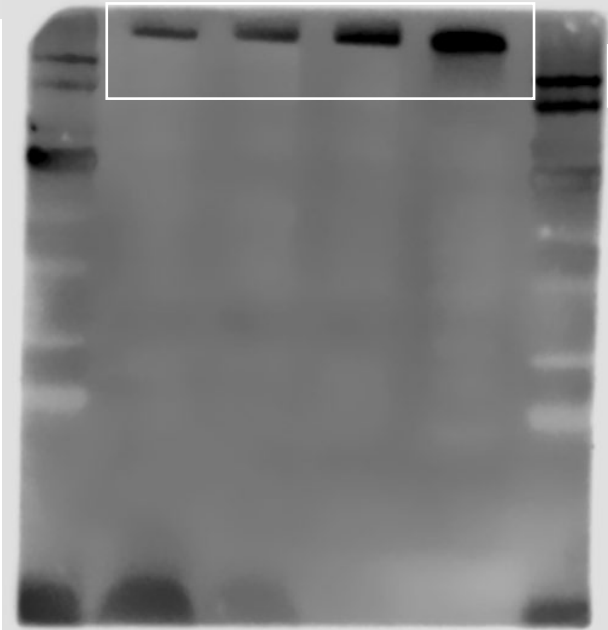

Adamts5

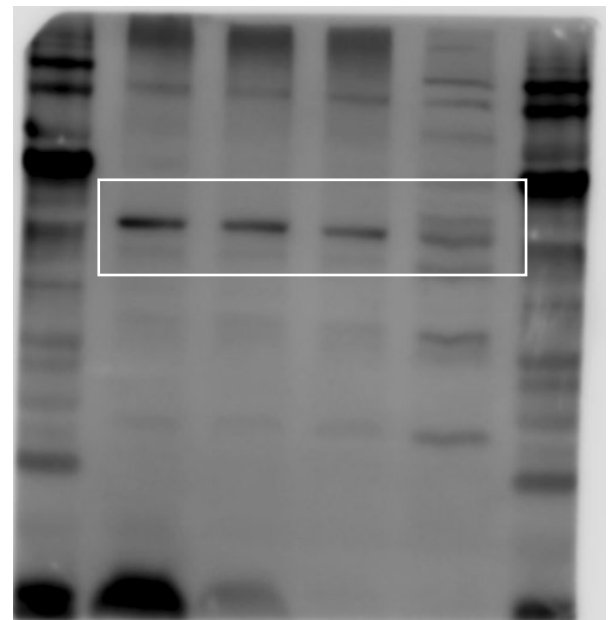

GAPDH

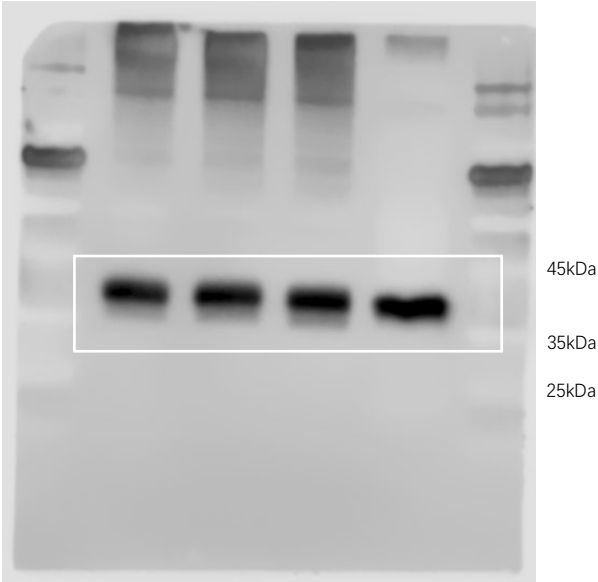

Supplement: Supplementary file 2 [file DataSheet1.PDF]
